# Geographic Expansion of Oropouche Fever Into Non‐Endemic Regions of Brazil: Implications for Health Surveillance

**DOI:** 10.1002/jmv.70744

**Published:** 2025-12-03

**Authors:** Rafael Pedro de Souza Nascimento, Paula Esbaltar de Oliveira, Adeilton Gonçalves da Silva Junior, Riana Oliveira Fernandes Amorim, Cleison Keulys dos Santos Silva, Daniela Silva Santos, Lethícia Lohane Almeida Barros, Hernany Fabricio de Novaes Menezes, Anderson da Costa Armstrong, Rodrigo Feliciano do Carmo, Carlos Dornels Freire de Souza

**Affiliations:** ^1^ College of Medicine, Federal University of the São Francisco Valley (UNIVASF) Petrolina Pernambuco Brazil; ^2^ Postgraduate Program in Family Health, Oswaldo Cruz Foundation (Fiocruz), Research CNPq N2 Rio de Janeiro Brazil

**Keywords:** Arbovirus infections. Disease outbreaks, emerging infectious diseases, non‐endemic transmission, Oropouche fever

## Abstract

Oropouche fever is an arboviral disease caused by the Oropouche virus (OROV; Peribunyaviridae family), first identified in Brazil in 1960. This study aims to describe the epidemiological profile and spatio‐temporal dispersion of Oropouche fever cases across Brazil in 2024. The variables analyzed were sex, age, transmission area, macroregion, and federative unit. A simple descriptive analysis (absolute frequencies and proportions) was performed. Data were extracted from the Oropouche Fever Epidemiological Panel and the Brazilian Institute of Geography and Statistics (IBGE). The analysis revealed 13,786 reported cases (6.80/100,000 inhabitants), with a predominance of males (52.6%; *n* = 7247). The North region presented the highest incidence (41.60%; *n* = 5732; 33.03/100,000 inhabitants). Incidence peaks during early (weeks 5–6) and late 2024 (week 52) were identified, with heterogeneous regional spread reflecting a possible outbreak progression from the North to Northeast and subsequently Southeast. Espírito Santo state recorded the highest national incidence (151.60/100,000 inhabitants; 42.20% of cases; *n* = 5812). The expansion of OROV into non‐endemic regions highlights an emerging public health threat. Urgent, systematic measures are required to strengthen Brazil's health surveillance system, ensuring timely and effective responses to mitigate further geographic spread.

## 1 Introduction

Oropouche fever is an arboviral disease caused by the Oropouche virus (OROV; Peribunyaviridae family), first isolated in Trinidad and Tobago in 1955 [1] and subsequently identified in Brazil in 1960 [2]. Since its initial detection, recurrent outbreaks have been documented across northern and northeastern Brazilian states. The first major epidemic occurred in 1961 in Pará, with ~11,000 reported cases, followed by subsequent outbreaks in Acre, Amazonas, Maranhão, and other northern states [3]. Oropouche fever infection outbreaks have been also reported in other Latin American countries, including Peru, Colombia, Ecuador and Venezuela [4]. Its occurrence often coincides with outbreaks of other clinically similar arboviral diseases, complicating differential diagnosis [1].

Globally, over 500,000 OROV infections have been reported, most of them in the Americas region [1]. In 2024 alone, approximately 16,000 cases were recorded across the American continent, with Brazil accounting for more than 80% of these infections; the remaining cases were distributed among Bolivia, Colombia, Cuba, Peru and Dominican Republic [5]. Recent evidence indicates that the current expansion of OROV into extra‐Amazonian regions is being driven predominantly by the reassorted M1L2S2 lineage, which differs substantially from the historical Amazonian genotypes. This lineage has been associated with large‐scale transmission in the Atlantic Forest biome, particularly in Espírito Santo, where strains belonging to the 2022–2024 Amazon lineage were recently identified [6].

As a notifiable disease in Brazil [2], surveillance data revealed approximately 1,000 confirmed human cases occurring between 2023 and early 2024 in Amazonas state. In February 2024, the Pan American Health Organization (PAHO) issued an alert urging member states to enhance surveillance of Oropouche fever cases [7]. Importantly, the emergence of severe clinical presentations coincided with this genomic shift: in 2024, the first confirmed fatal cases of OROV infection were documented in non‐endemic areas of Brazil [8], and subsequent investigations have provided further insight into the pathogenesis of severe OROV disease caused by contemporary reassortant strains [9]. By the end of 2024, Brazil reported over 13,000 cases nationwide [10].

Moreover, the first neuroinvasive infection attributed to the M1L2S2 lineage was recently reported during the country's largest recorded epidemic [11]. Together, these findings underscore the epidemiological and clinical relevance of the emergent reassorted lineage and highlight the need for updated surveillance and genomic monitoring across Brazil.

## 2 Materials and Methods

### 2.1 Study Design, Population and Period

The present study is an ecologic study involving all notified cases of Oropouche fever in Brazil during 2024.

### 2.2 Study Setting

Brazil comprises five macroregions (North, Northeast, Southeast, South, and Central‐West) and 27 federative units (including the Federal District). Brazil has a total area of around 8.5 million km², the fifth‐largest globally [12].

### 2.3 Data Source and Variables

Data were extracted from the publicly available Oropouche Fever Epidemiological Panel (https://www.gov.br/saude/pt-br/assuntos/saude-de-a-a-z/o/oropouche/painel-epidemiologico) [10]. Population data used for incidence calculations were sourced from the Brazilian Institute of Geography and Statistics (IBGE).

The variables included were: sex (male and female), age (0–4; 5–9; 10–19; 20–59; ≥ 60 years), transmission area (endemic and non‐endemic regions), macroregion (North, Northeast, Southeast, Central‐West and South), state and epidemiologic week of diagnosis (weeks 1 to 52). The primary morbidity indicator used was the incidence rate (IR), calculated as:

It was assumed that the population of each location remained constant throughout the epidemiologic weeks. Age categories were defined as 0–4, 5–9, 10–19, 20–59, and ≥ 60 years. The broad 20–59 year category was selected to encompass the entire young adult and middle‐aged population.

### 2.4 Statistical Analysis

Descriptive analyses were performed using absolute frequencies and proportions. Temporal trends and spatial distributions were visualized via time‐series graphs and maps generated using: JASP (version 0.16.1.0, University of Amsterdam/Amsterdam, The Netherlands) and QGIS (version 2.14.11, Open Source Geospatial Foundation (OSGeo), Beaverton, OR, USA).

### 2.5 Ethical Aspects

As this study used aggregated, publicly available data, ethics approval was waived per Resolution 510/2016 of Brazil's National Research Ethics Committee (CONEP).

## 3 Results

The analysis revealed 13,786 reported cases (6.80/100,000 inhabitants) in 2024, with a predominance of males (52.6%; *n* = 7247; 7.36/100,000 inhabitants). The North region presented the highest incidence (41.60%; *n* = 5732; 33.03/100,000 inhabitants), followed by the Southeast (45.40%; *n* = 6258; 7.40/100,000 inhabitants). The Central‐West had the lowest incidence (0.4%; *n* = 53; 0.33/100,000 inhabitants) (Table 1).

**Table 1:** Epidemiological characteristics of Oropouche fever cases in Brazil, 2024.

Variables	% (*n*)	Incidence rate/100.000
Sex		
Male	52.60% (7247)	7.36
Female	47.36% (6539)	6.26
Blank/Ignored	0.04% (5)	—
Age		
0–4	0.70% (94)	0.74
5–9	2.10% (284)	2.10
10–19	12.50% (1715)	6.11
20–59	71.21% (9817)	8.43
≥ 60	13.60% (1870)	5.82
Blank/Ignored	0.04% (6)	—
Transmission area^a^		
Endemic region^b^	42.10% (5803)	33.44
Non endemic region^c^	57.90% (7983)	4.30
Geographic region		
North	41.60% (5732)	33.03
Northeast	11.22% (1547)	2.83
Central‐West	0.4% (53)	0.33
Southeast	45.4% (6258)	7.40
South	1.42% (196)	0.66
		
** Total**	100% (13,786)	6.80

a Only states with autochthonous cases were included.

b Endemic region: Acre, Amapá, Amazonas, Pará, Rondônia, Roraima, Tocantins.

c Non‐endemic regions: Bahia, Ceará, Maranhão, Paraíba, Pernambuco, Piauí, Sergipe, Alagoas, Espírito Santo, Minas Gerais, Rio de Janeiro, São Paulo, Santa Catarina, Mato Grosso, Mato Grosso do Sul.

**Figure 1:**
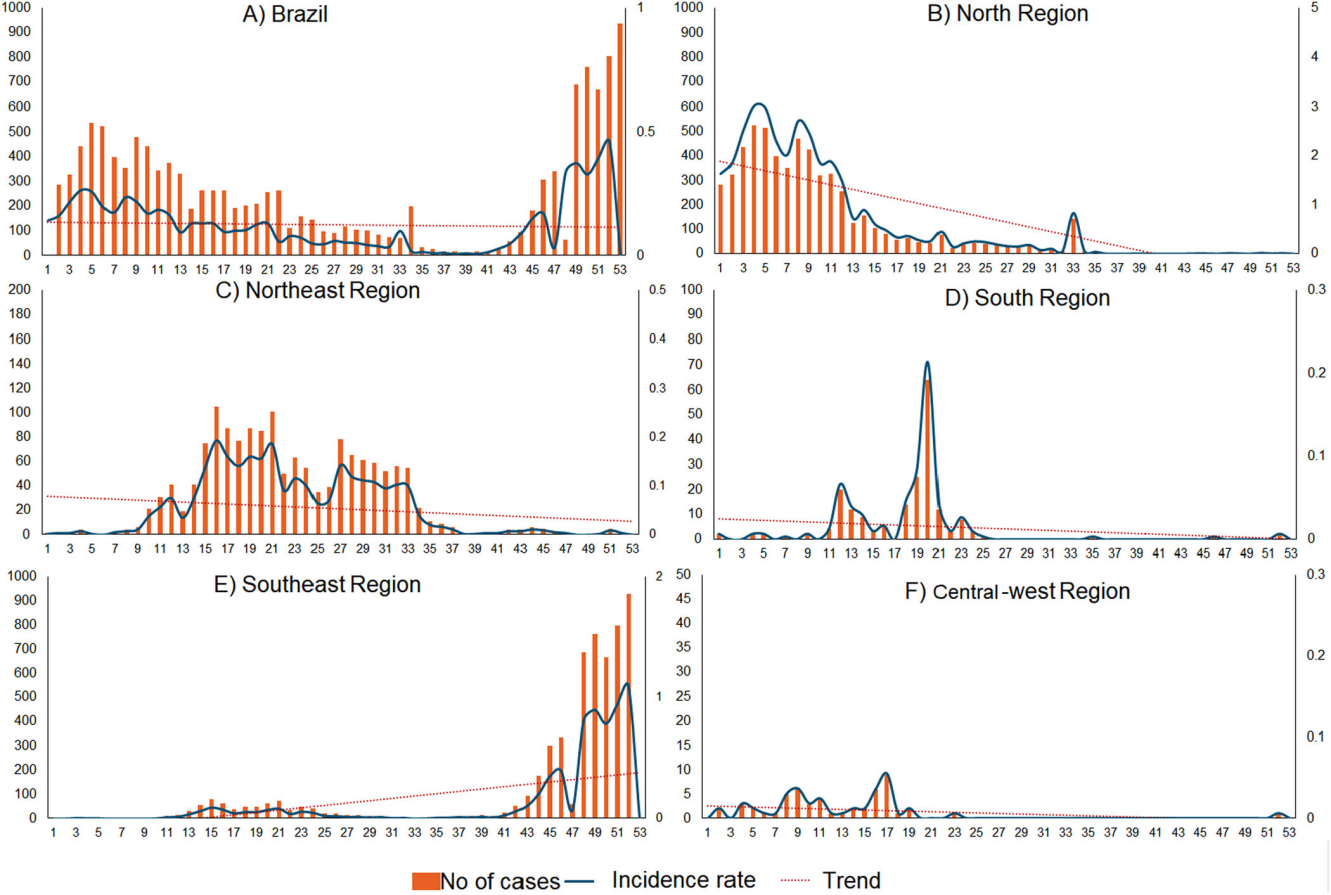
Trends of Oropouche fever incidence rate and number of cases in Brazil, 2024.

**Figure 2:**
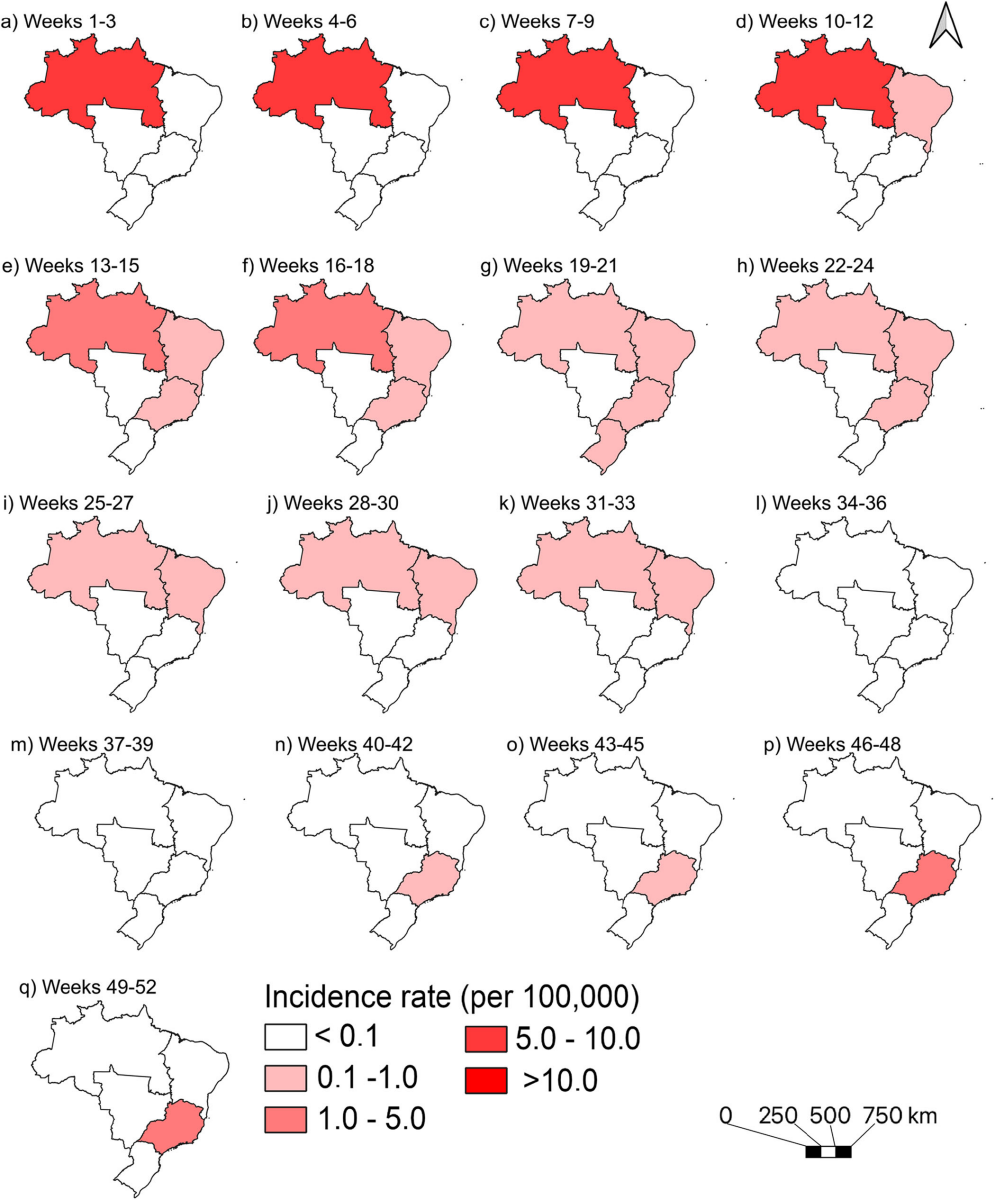
Geographic distribution of Oropouche fever incidence rates across Brazilian macroregions, 2024.

Cases occurred year‐round, with two distinct peaks: early (weeks 5 and 6) and late 2024 (weeks 49 to 52), with heterogeneities among regions. The North region peaked earlier, during weeks 4 (*n* = 537) and 8 (*n* = 469), then declined. Its annual incidence (33.03/100,000) was 4.5 times higher than the Southeast's (7.40/100,000). After the North's decline, cases rose in the Northeast (weeks 15 to 33), shifted to the Southeast (week 41 onward). The Central‐West and South had only sporadic and isolated cases of the disease (Figures 1 and 2).

**Figure 3:**
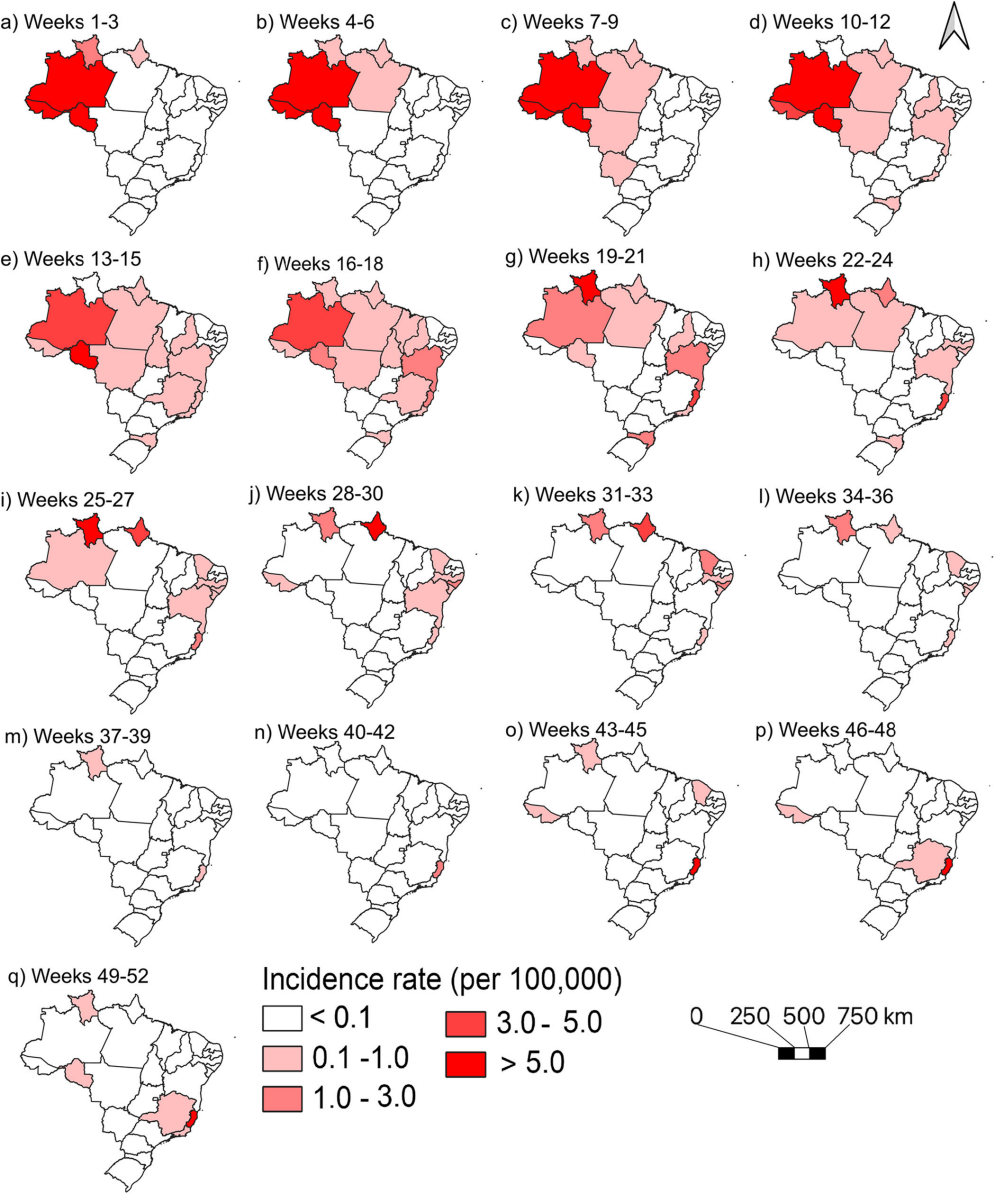
Geographic distribution of Oropouche fever incidence rates across Brazilian states, 2024.

Six states exceeded Brazil's incidence rate (6.80/100,000), five in the North. Espírito Santo, despite being in the Southeast, was the most affected state (151.60/100,000; 42.20%; *n* = 5812), followed by Rondônia (105.40/100,000; 12.10%; *n* = 1667). Other states with high burdens included Amazonas (80.50/100,000), Roraima (45.40/100,000), and Acre (34.50/100,000). Outside endemic areas, only Espírito Santo surpassed the national rate (Figure 3).

Despite bordering northern states, the Central‐West region did not show high incidence rates, with 5, 39 and 8 cases reported in Goiás, Mato Grosso and Mato Grosso do Sul, respectively, and only one case in the Federal District. Conversely, Maranhão (0.60/100,000 inhabitants), Paraíba (0.33/100,000 inhabitants) and Rio Grande do Norte (0.10/100,000 inhabitants) presented lower rates compared to neighboring states. In the South, Santa Catarina (2.40/100,000) showed a marked contrast with Rio Grande do Sul (0.02/100,000) and Paraná (0.10/100,000) (Figure 3).

## 4 Discussion

This study aimed to describe the epidemiological profile and spatio‐temporal dispersion of Oropouche fever cases across Brazil in 2024. Cases predominated among males and in the North region (notably Amazonas and Rondônia). Non‐endemic areas, particularly the Southeast (carried by Espírito Santo), also exhibited significant incidence, with distinct regional peaks in case notifications.

The observed epidemiological patterns are consistent with a 2022 cohort study conducted in border regions of the Amazon, in which 59.26% of cases occurred among young adults [13]. The geographic findings corroborate a 2023 review on the epidemiology and molecular biology of OROV, which highlights the North and Northeast as historical epicenters of OROV transmission, reporting the highest incidences since the virus was first described [14]. More recently, however, in 2024, the Southeast has emerged as a region of incresing epidemiological relevance.

Urbanization, deforestation [15, 16], and the effects of climate change [15] are known to amplify the proliferation of the biting midge vector *Culicoides paraensis* [17], thereby increasing the risk of re‐emergence and expansion of the virus in the Amazon region [15]. Genetic factors should also be considered; a review study published in 2017 suggests that genetic shifts in OROV strains may have enhanced virulence or host adaptability [18]. These factors collectively contribute to the rising case burden in Brazil.

Beyond ecological and demographic drivers, the contemporary expansion of OROV appears to be strongly influenced by the dissemination of the reassorted M1L2S2 genotype, which has rapidly replaced earlier circulating strains in several regions. This lineage has been implicated not only in widespread transmission but also in the emergence of more severe disease phenotypes. In 2024, Brazil documented the first fatal OROV infections in a non‐endemic region, a clinical development not previously observed in over six decades of virus circulation [8]. Subsequent analyses have begun to unravel possible mechanisms underlying these severe outcomes, suggesting altered viral–host interactions and enhanced pathogenic potential in reassortant strains [9]. Such findings reinforce the likelihood that genomic evolution is contributing materially to the observed changes in epidemiological dynamics.

This investigation recorded an increase in OROV cases in the Northeast, Central‐West and Southeast, which are considered non‐endemic. Regarding the occurrence of cases in the Central‐West and Southeast, a 2023 review on the Oropouche Fever dynamics pointed out that, since the early 2000s, OROV has been detected in nonhuman primates in the Southeast. Additionally, intensified human migration, along with improvements in transportation and tourism infrastructure, may have facilitated the spread of the virus to non‐endemic regions [14].

The Northeast region and key states—notably Espírito Santo (Southeast) and Santa Catarina (South)—emerged as significant foci of extra‐Amazonian transmission. These areas were reported in another study [19], and likely became hotspots due to a combination of immunologically susceptible populations and demographic factors (e.g., high population density, which facilitates efficient viral spread—a mechanism consistent with prior findings) [14]. Additionally, avian‐mediated viral dispersal via migratory birds has been proposed as another potential dissemination pathway [15].

The clinical spectrum associated with the current epidemic has also broadened. Most notably, a neuroinvasive OROV infection associated with the M1L2S2 lineage was recently confirmed in a patient living with HIV in extra‐Amazonian Brazil, marking the first documented case of central nervous system involvement by this emergent reassortant strain [11]. This case occurred amid the largest OROV epidemic ever recorded in the country and raises important questions regarding host susceptibility, viral determinants of neurotropism, and the potential for more severe disease in high‐risk populations. As such, these observations further emphasize the significance of incorporating genomic and clinical data into routine surveillance frameworks, particularly in regions undergoing recent viral introduction.

Since May 2024, PAHO has issued alerts regarding unprecedented autochthonous transmission in extra‐Amazonian regions [20]. Given the virus's demonstrated adaptability [14], these warnings emphasized the urgent need for enhanced health surveillance and vector control measures [20]. A subsequent PAHO update in September 2024 further reinforced this position, highlighting the critical importance of preventive surveillance, improved laboratory diagnostics, and optimized clinical management [5].

There are, so far, no approved vaccines or specific therapeutics for OROV infection. In response, the Brazilian government has adopted compulsory disease notification policies to enable rapid investigation and transmission chain interruption [21]. Complementing these measures, environmental preservation strategies targeting the disease's ecological determinants [15, 16] should be prioritized to control the current outbreak and mitigate future epidemic risks.

Despite rigorous methodological care, this study has limitations. First, secondary surveillance data may be subject to underreporting, particularly given: (1) symptom overlap with co‐circulating arboviruses [20, 22, 23], and (2) healthcare system strain during peak transmission periods [23]. Second, the lack of epidemiological studies on OROV restricts our ability to conduct robust temporal or spatial comparisons of disease evolution.

## 5 Conclusion

A high incidence was observed in 2024, concentrated in the North region among adult males, followed by a spatial shift characterized by declining incidence in the North and increasing transmission in the Southeast and Northeast, along with sporadic cases in the South and Central‐West.

These findings underscore the urgent need for a tiered public health response: (1) strengthening integrated surveillance networks with real‐time data sharing across all government levels (municipality, state and federation); (2) expanding diagnostic capacity through decentralized testing and active case‐finding; and (3) optimizing resource allocation to improve outbreak containment. These measures are required to strengthen Brazil's health surveillance system, ensuring timely and effective responses to mitigate further geographic spread.

## Author Contributions

Conceptualization: Carlos Dornels Freire de Souza, Paula Esbaltar de Oliveira, Rafael Pedro de Souza Nascimento, Adeilton Gonaçalves da Silva Junior, Riana Oliveira Fernandes Amorim, Cleison Keulys dos Santos Silva, Daniela Silva Santos, Lethícia Lohane Almeida Barros, Hernany Fabricio de Novaes Menezes, Anderson da Costa Armstrong and Rodrigo Feliciano do Carmo. Formal analysis: Carlos Dornels Freire de Souza, Paula Esbaltar de Oliveira, Rafael Pedro de Souza Nascimento, Adeilton Gonaçalves da Silva Junior, Riana Oliveira Fernandes Amorim, Cleison Keulys dos Santos Silva, Daniela Silva Santos, Lethícia Lohane Almeida Barros, Hernany Fabricio de Novaes Menezes, Anderson da Costa Armstrong and Rodrigo Feliciano do Carmo. Writing—original draft preparation: Carlos Dornels Freire de Souza, Paula Esbaltar de Oliveira, Rafael Pedro de Souza Nascimento, Adeilton Gonaçalves da Silva Junior, Riana Oliveira Fernandes Amorim, Cleison Keulys dos Santos Silva, Daniela Silva Santos, Lethícia Lohane Almeida Barros, Hernany Fabricio de Novaes Menezes, Anderson da Costa Armstrong and Rodrigo Feliciano do Carmo. Writing—review and editing: Carlos Dornels Freire de Souza, Paula Esbaltar de Oliveira, Rafael Pedro de Souza Nascimento, Adeilton Gonaçalves da Silva Junior, Anderson da Costa Armstrong and Rodrigo Feliciano do Carmo. All the authors have read and agreed to the published version of the manuscript.

## Conflicts of Interest

The authors declare no conflicts of interest.

## Data Availability

The data that support the findings of this study are publicly available on the Oropouche Fever Epidemiological Panel (https://www.gov.br/saude/pt-br/assuntos/saude-de-a-a-z/o/oropouche/painel-epidemiologico).
